# Biosynthesis of the redox cofactor mycofactocin is controlled by the transcriptional regulator MftR and induced by long-chain acyl-CoA species

**DOI:** 10.1016/j.jbc.2021.101474

**Published:** 2021-12-09

**Authors:** Aigera Mendauletova, John A. Latham

**Affiliations:** Department of Chemistry and Biochemistry, University of Denver, Denver, Colorado, USA

**Keywords:** mycobacteria, gene regulation, coenzyme A, fatty acid metabolism, biosynthesis, mycofactocin, 6-FAM, 6-carboxyfluorescein, 7H9, Middlebrook 7H9, BGC, biosynthetic gene cluster, CFU, colony-forming unit, FP, fluorescence polarization, HTH, helix–turn–helix, ITC, isothermal titration calorimetry, MFT, mycofactocin, Msmeg, *Mycobacterium smegmatis* mc2155, MTB, *Mycobacterium tuberculosis*, OA, oleic acid, Omft, *mft* operator, PDB, Protein Data Bank, P_mft_, *mft* promoter, PMFT, premycofactocin, qPCR, quantitative PCR, RiPP, ribosomally synthesized and post-translationally modified peptide, TFR, TetR family regulator

## Abstract

Mycofactocin (MFT) is a ribosomally synthesized and post-translationally-modified redox cofactor found in pathogenic mycobacteria. While MFT biosynthetic proteins have been extensively characterized, the physiological conditions under which MFT biosynthesis is required are not well understood. To gain insights into the mechanisms of regulation of MFT expression in *Mycobacterium smegmatis* mc^2^155, we investigated the DNA-binding and ligand-binding activities of the putative TetR-like transcription regulator, MftR. In this study, we demonstrated that MftR binds to the *mft* promoter region. We used DNase I footprinting to identify the 27 bp palindromic operator located 5′ to *mftA* and found it to be highly conserved in *Mycobacterium tuberculosis*, *Mycobacterium bovis*, *Mycobacterium ulcerans*, and *Mycobacterium marinum*. To determine under which conditions the *mft* biosynthetic gene cluster (BGC) is induced, we screened for effectors of MftR. As a result, we found that MftR binds to long-chain acyl-CoAs with low micromolar affinities. To demonstrate that oleoyl-CoA induces the *mft* BGC *in vivo*, we re-engineered a fluorescent protein reporter system to express an MftA–mCherry fusion protein. Using this mCherry fluorescent readout, we show that the *mft* BGC is upregulated in *M. smegmatis* mc^2^155 when oleic acid is supplemented to the media. These results suggest that MftR controls expression of the *mft* BGC and that MFT production is induced by long-chain acyl-CoAs. Since MFT-dependent dehydrogenases are known to colocalize with acyl carrier protein/CoA-modifying enzymes, these results suggest that MFT might be critical for fatty acid metabolism or cell wall reorganization.

Organic redox cofactors are essential for life. While classic flavins and nicotinamides are widely distributed across all domains of life, nature has also evolved niche cofactors in subsets of life domains. For example, in *Actinobacteria*, coenzyme F_420_ is commonly used in place of FMN in enzymes associated with carbon fixation (1) and oxidation of secondary alcohols (2). The importance of niche cofactors has long been recognized; however, detailed understanding about their biosynthesis and physiological uses has been lagging. One class of niche cofactors is derived from ribosomally synthesized and post-translationally modified peptides (RiPPs) (3). To achieve their mature form, the genetically encoded RiPP precursor peptide undergoes significant post-translational modifications by diverse families of tailoring enzymes (4, 5, 6). Following synthesis by the ribosome, modifying enzymes process the precursor peptide into the mature redox cofactor. Currently, there are two known RiPP-derived redox cofactors, pyrroloquinoline quinone (7), which has been well characterized, and mycofactocin (MFT), which was recently discovered.

The MFT biosynthetic gene cluster (BGC) consists of *mftABCDEF* (Fig. 1*A*) and is highly conserved in mycobacteria, including pathogens, such as *Mycobacterium tuberculosis* (MTB), *Mycobacterium ulcerans*, *Mycobacterium avium*, and *Mycobacterium bovis* (8). As shown in Figure 1*B*, MFT biosynthesis starts with the MftC-catalyzed oxidative decarboxylation of the C-terminal Tyr forming MftA∗∗ (9, 10) and the subsequent formation of a C–C bond resulting in a lactam derived from penultimate Val, MftA∗ (11). Both reactions are dependent upon the RiPP recognition element MftB, which binds MftA and delivers it to MftC (9). Next, MftE hydrolyzes MftA∗, forming 3-amino-5-[(p-hydroxyphenyl)methyl]-4,4-dimethyl-2-pyrrolidinone (12). Following cleavage, MftD catalyzes the FMN-dependent oxidation of the 3-amino group, resulting in an α-keto-amide moiety within the lactam, forming premycofactocin (PMFT; Fig. 1*B*) (13). Finally, a recent metabolomics analysis has indicated that MftF glycosylates PMFT with up to eight β1–β4 glucans, forming mature MFT (14).

**Figure 1:**
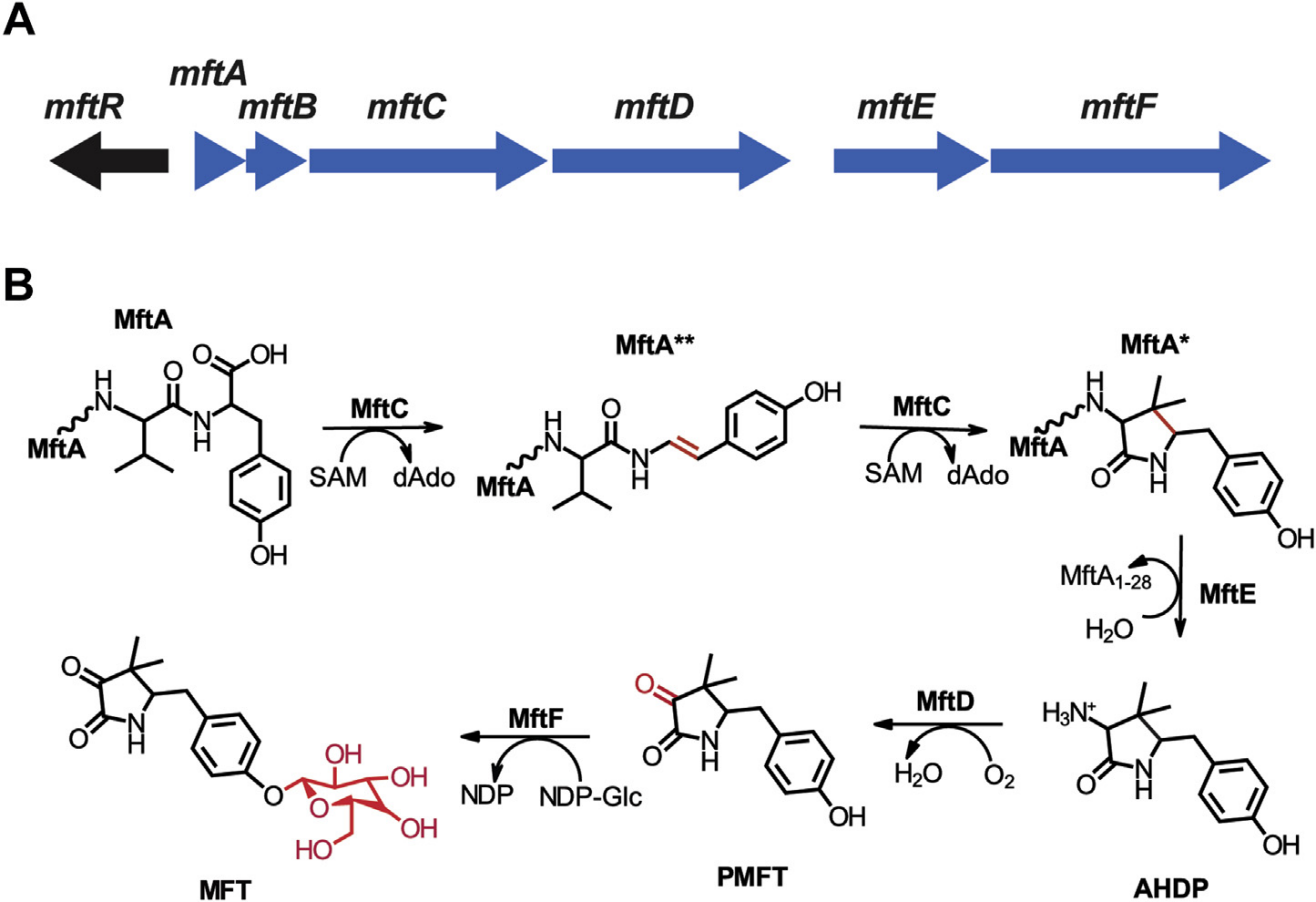
**Mycofactocin (MFT) biosynthetic gene cluster.***A*, a schematic depiction of the gene organization of the MFT biosynthetic gene cluster. *B*, the MFT biosynthesis model; the enzyme modifications are highlighted in *red*.

In addition to *mft* genes, three different dehydrogenase families (TIGR03971, TIGR03989, and TIGR04266) are found in genomes only when the *mft* BGC is present (15). These MFT-dependent dehydrogenases have been shown to sequester NADH within their active sites and therefore require an additional electron acceptor, presumably MFT, to oxidize NADH for further catalytic turnover (16). In support of this, knockouts of the *mft* genes in *Mycobacterium smegmatis* mc^2^155 (Msmeg) led to the inability of the organism to maintain homeostasis of cellular NAD^+^/NADH pools and its inability to metabolize methanol and ethanol (17, 18). The failure of the knockouts to metabolize primary alcohols is likely because of the MFT-dependent alcohol dehydrogenase, Msmeg_6242, being trapped in a reduced state in the absence of MFT. More recently, a study demonstrated that *mftD*, and thus MFT, is required for Mtb survival *in vitro* and *in vivo* under hypoxic conditions (19). However, until recently, direct evidence demonstrating MFT is a redox cofactor was nonexistent. This changed when it was shown that both PMFT and MFT are capable of oxidizing MFT-dependent dehydrogenases *in vitro* (13, 14). Despite knowing the structure, biosynthesis, and redox attributes of MFT, information about physiological processes that require MFT has been lagging.

One way to address the physiological dependence on MFT is to understand how MFT biosynthesis is regulated. Currently, it is thought that putative TetR-like protein MftR is a regulator of MFT biosynthesis (20, 21). In general, TetR family regulators (TFRs) are transcription repressors and implicated in the regulation of efflux pumps (22), antibiotic biosynthesis (23), the tricarboxylic acid cycle (24), biofilm formation (25), and quorum sensing molecules (26). TFRs are functional dimers that contain a DNA-binding domain and a regulatory domain (27). The DNA-binding domain consists of a helix–turn–helix (HTH) motif that binds to a DNA operator sequence (28). The regulatory domain consists of a binding pocket that specifically interacts with a variety of compounds, such as tetracycline (29), biotin (30), fatty acid CoAs (31, 32), flavonoids (33), and cell–cell signaling molecules (34), depending on the system. MftR regulation of the *mft* BGC is supported by bioinformatics, which suggests that the gene proximity of *mftR* and its arrangement to the *mft* BGC is indicative of regulatory control of MFT biosynthesis (35). In addition, a transcriptomics study of macrophage samples infected with MTB showed that upregulation of the MftR homolog, Rv0691c, led to the repression of *mftB*, *mftC*, and *mftD* (36). Currently, the specific DNA operator sequence that MftR recognizes, its regulatory role over the *mft* BGC in Msmeg, and the conditions that MftR could regulate MFT biosynthesis are unknown.

Here, we report that *msmeg_1420*, annotated as MftR, is a transcriptional repressor of the *mft* BGC in Msmeg. We found that MftR binds a DNA sequence in the promoter region of the *mft* BGC. We mapped the 27 bp *mft* operator (O_mft_) by DNase I footprinting and measured dissociation constant (*K*_*d*_) of the MftR–O_mft_ complex by fluorescence anisotropy. We employed relative RT–quantitative PCR (qPCR) to demonstrate that overexpression of MftR results in the repression of *mft* genes in Msmeg. To determine under what conditions the biosynthesis of MFT might be induced, we employed EMSAs and isothermal titration calorimetry (ITC) to identify effectors of MftR. To demonstrate that identified effectors translate *in vivo*, we use an engineered fluorescence reporter system to show that effectors supplemented to growth media induces the expression of the *mft* BGC in Msmeg and quantify the induction relative to RT–qPCR. These findings provide a mechanism to understand the physiological conditions that MFT is regulated and thus required *in vivo*.

## Results

### Identifying and sequencing the MFT operator

**Figure 2:**
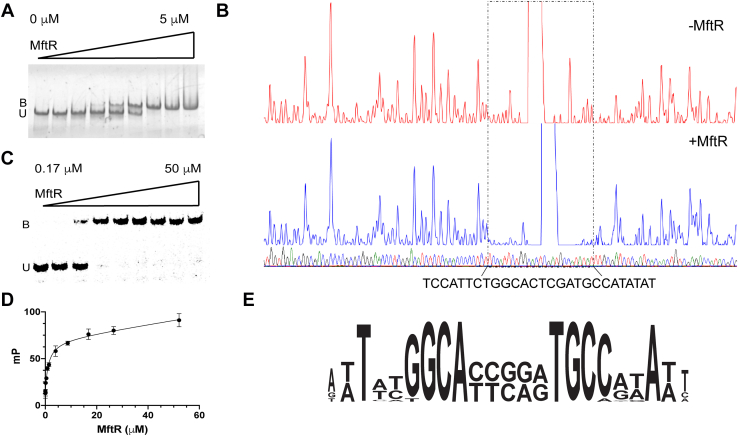
**Identification of the mycofactocin operator.***A*, a representative EMSA of the P_mft_–MftR complex. The P_mft_ region (0.5 μM) was mixed with increasing concentrations of MftR. The U and B represent unbound and bound fractions, respectively. Protein–DNA complexes were separated by electrophoresis on 5% polyacrylamide gel and imaged using GelRed nucleic acid stain. The assays were performed in triplicate, producing similar results. *B*, DNase I footprinting assay to determine the binding site of P_mft_ to MftR. Top fluorogram shows control reaction of P_mft_ 350 ng with no protein added. Upon the addition of MftR (2 μg), the distinct binding area was determined and is shown in the bottom fluorogram. The 27 base pairs sequence (O_mft_) on the P_mft_–MftA was confirmed to be the binding region responsible for the interaction with MftR. *C*, an EMSA validating that MftR binds to O_mft_. Fluorescein-labeled O_mft_ (0.5 μM) was mixed with increasing concentrations of MftR. The U and B represent unbound and bound fractions, respectively. Protein–DNA complex was separated by electrophoresis on 5% polyacrylamide gel and imaged using FAM excitation and emission wavelengths. The assays were performed in triplicate, producing similar results. *D*, a representative fluorescence polarization binding assay showing the change in polarization of FAM–O_mft_ as a function of MftR concentration. All assays were performed in triplicate, and the average (*filled circles*) and standard deviations (*error bars*) are shown with the nonlinear fit (*line*). *E*, a WebLogo representation of the O_mft_ region showing the palindromic sequence found in 83 species of *Mycobacterium* and *Mycolicibacterium*. FAM, carboxyfluorescein; P_mft_, *mft* promoter.

To provide evidence that MftR is a regulator of MFT biosynthesis, we ran a series of EMSAs to demonstrate that MftR binds to the MFT promoter region. To begin with, recombinant His-tagged Msmeg MftR was purified from *Escherichia coli* (Fig. S1) and the 565 bp promoter region (P_mft_) between −470 to +95 relative to MftA was PCR amplified. Next, EMSAs were carried out in triplicate with a fixed concentration of the PCR-amplified P_mft_ and varying concentrations of MftR. As shown in Figure 2*A*, the addition of MftR to unlabeled P_mft_ resulted in a single impeded band in a concentration-dependent manner with an estimated *K*_*d*_ ∼ 0.6 μM. This result indicates that at least a single binding site of MftR with at least one binding affinity is present in the P_mft_ regulatory region.

To determine the exact location of the MftR-binding site in the *mft* regulatory region, DNase I protection assays were performed using a P_mft_ DNA probe labeled with 6-carboxyfluorescein (6-FAM), in the presence and absence of MftR. As shown in Figure 2*B*, MftR protected a single region extending from −79 to −53 from DNase I digestion. The shift in DNase I hypersensitivity by three nucleotides when MftR is present suggests the establishment of new contacts being made to and/or a modification of the DNA structure. To validate this finding, the MftR protected sequence was synthesized with a 6-FAM label and used in a subsequent EMSA. As shown in Figure 2*C*, increasing concentrations of MftR resulted in a single concentration-dependent impeded band, consistent with the original EMSA with P_mft_. Controls with a “cold” competitive ligand and with a nonspecific DNA sequence (Fig. S2) suggest that the interaction between MftR and the identified region is specific. In addition, fluorescence anisotropy experiments were carried out to estimate the binding affinity between MftR and the 6-FAM-labeled 27 bp sequence. Consistent with EMSAs, increasing concentrations of MftR resulted in a concentration-dependent change in polarization, which upon fitting three independent experiments to a single-site binding model, led to an observed *K*_*d*_ of 1.3 ± 0.6 μM (Fig. 2*D*). As a result, we propose that the MFT operator (O_mft_) sequence includes at least one MftR binding motif within the sequence 5′-TCCATTCTGGCACTCGATGCCATATAT (Fig. 2*E*).

**Figure 3:**
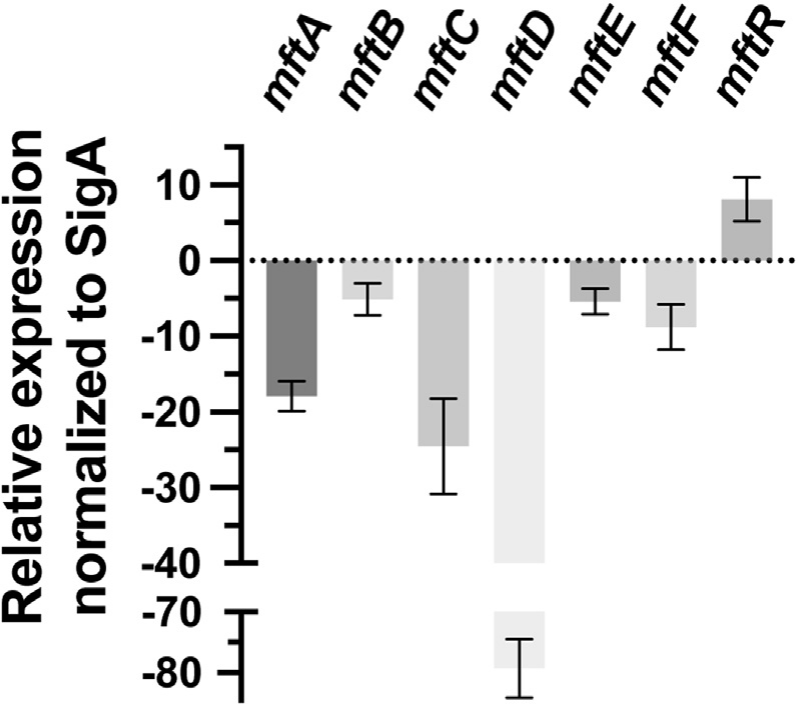
**Overexpression of MftR downregulates the mycofactocin (MFT) biosynthetic gene cluster (BGC).** A qRT–PCR–based quantification of the MFT BGC in *Mycobacterium smegmatis* grown in 7H9–ADC. The relative expression levels of the differentially expressed genes were compared between the bacterial strains wildtype *M. smegmatis* and *M. smegmatis* transformed with pMftR+. 7H9, Middlebrook 7H9; qRT, quantitative RT.

Next, we employed real-time qRT–PCR analysis to demonstrate that MftR regulates the *mft* BGC *in vivo*. We measured the transcript levels of *mftA-F* and *mftR* in wildtype Msmeg and compared them to the transcript levels in Msmeg harboring a mycobacterial expression vector consisting of *mftR* under the control of the constitutive expression promoter P_smyc_ (pMftR+). Overproduction of *mftR* in the expression strain, as compared with wildtype Msmeg, was confirmed by qRT–PCR analysis, which revealed that the *mftR* transcript abundance was increased by approximately fivefold (Fig. 3). Conversely, overproduction of *mftR* led to reduced transcript levels in all *mft* biosynthetic genes. Notably, the transcript levels of *mftA* and *mftC* were reduced by ∼15-fold and ∼20-fold, respectively. However, the most remarkable change in transcript levels was that of *mftD*, which was reduced nearly 80-fold. The transcript levels for *mftB*, *mftE*, and *mftF* were also decreased however, to a lesser extent (<10-fold). Taken together with the EMSAs, DNA footprinting, and fluorescence anisotropy experiments, MftR is a regulator of MFT biosynthesis in Msmeg.

### Expanding MftR role in mycobacteria

We examined if the position and sequence of O_mft_ in the *mft* promoter region is similar in MTB since the organism encodes for the *mft* operon (*rv0691a–rv0696*) and a MftR homolog (*rv0691c*, 69% identity). To answer this question, we carried out a BLAST analysis of the identified O_mft_ sequence and the ∼500 bp *mft* promoter from MTB. Accordingly, we found a single well-aligned sequence with 85% conservation. Similar to Msmeg, the putative O_mft_ in MTB extends from −78 to −52 relative to the *mftA* start codon, with the assumption that the operator sequence is the same length. We expanded our search to the *Mycobacterium* and *Mycolicibacterium* genera using a customized BLAST analysis (expect = 1000; match/mismatch = 1, −1; gap costs = 0, 2). Under these conditions, 83 sequences were identified with sequence identities >84%. As shown in the WebLogo (37) depiction of the multiple sequence alignment of all sequences (Fig. 2*E*), we found that putative O_mft_ regions are highly conserved. In addition, our analysis identified a palindromic region consisting of the residues T-N_2_-GGCA-N_5_-TGCC-N_2_-A. Despite the apparent conservation of the palindrome, single nucleotide replacements within the sequence did not impede the ability of MftR to bind O_mft_ in fluorescence polarization (FP) or EMSAs (data not shown).

### Long-chain acyl-CoAs are effectors of MftR

**Figure 4:**
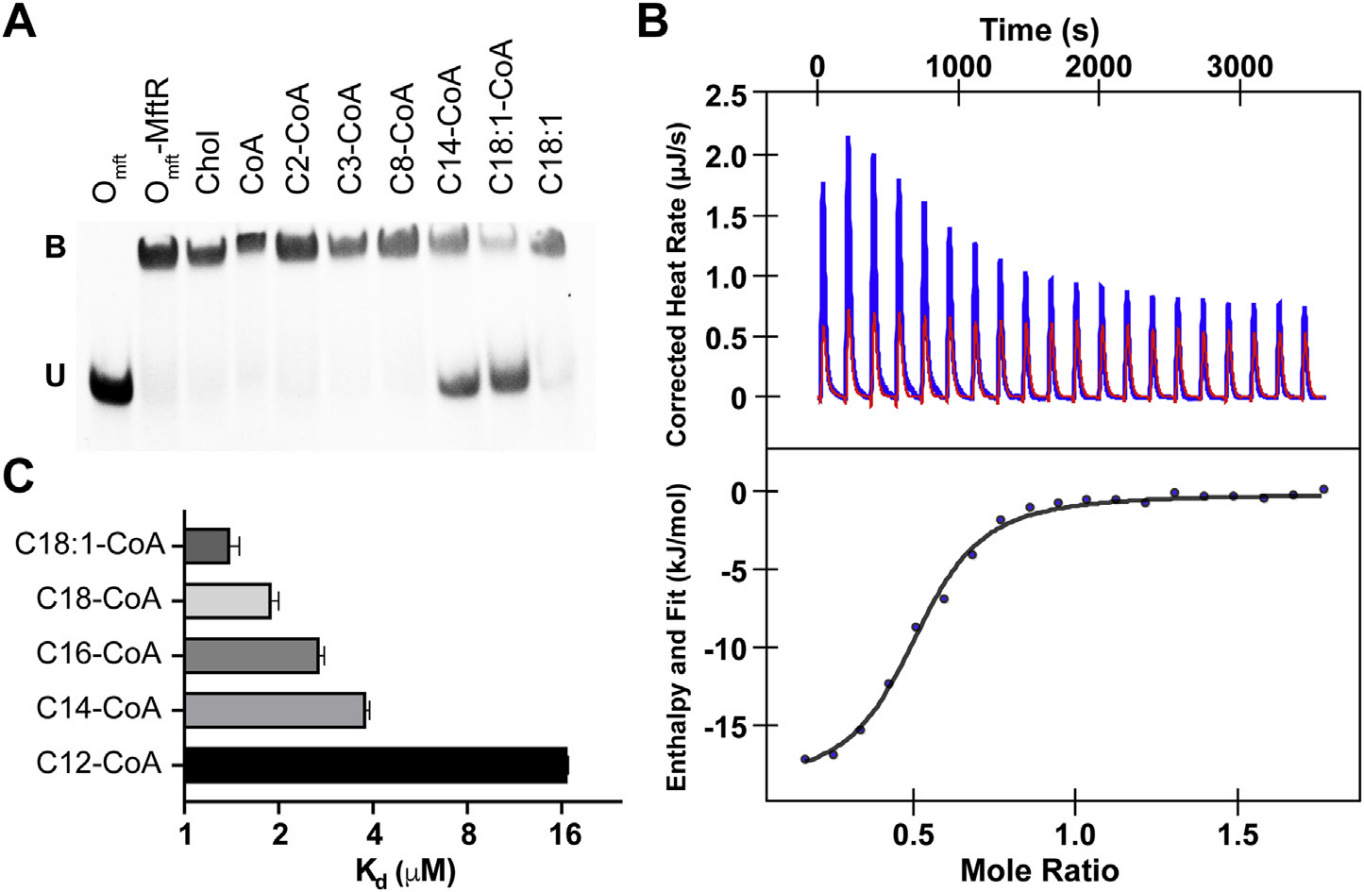
**Effectors of MftR.***A*, an EMSA used to screen effectors of MftR. The 6-FAM–labeled O_mft_ region (0.5 μM) was incubated with the MftR (5 μM) in the absence or the presence of effectors (100 μM). Protein–DNA complexes were separated by electrophoresis on 5% polyacrylamide gel. The assays were performed in triplicate, producing similar results. *B*, a representative isothermal titration calorimetry thermogram for the binding of the oleoyl-CoA to MftR regulator (*blue*) and the control of oleoyl-CoA into buffer (*red*). Each peak corresponds to the injection of 2.22 μl of 250 μM oleoyl-CoA into the cell containing 16 μM MftR or buffer. The integrated thermogram (*blue dots*) was fitted to a single-site binding model (*black line*) to determine the *K*_*d*_. The experiment was done in triplicate producing the similar results. *C*, a bar graph depicting the ITC measured *K*_*d*_s for various acyl-CoAs. Bars represent the mean of three independent experiments, and the error bars represent the standard deviation of the experiments. 6-FAM, 6-carboxyfluorescein; ITC, isothermal titration calorimetry; O_mft_, *mft* operator.

Next, we assessed which metabolites activate MftR and thus could induce MFT production. To do so, we carried out competitive EMSAs where FAM-labeled O_mft_ and MftR were incubated in the presence of potential effectors. We initially chose our effectors based on cholesterol catabolism, a process that putatively includes MFT biosynthetic genes (38). Despite the loose association of MFT to cholesterol catabolism, we did not observe DNA release by MftR in the presence of cholesterol (Fig. 4*A*, lane 3), propionyl-CoA (Fig. 4*A*, lane 6), succinyl-CoA (not shown), and acetoacetyl-CoA (not shown). Knowing that TFRs have a propensity to be activated by fatty acyl-CoAs (20), we expanded our effector screening to include short-chain, medium-chain, and long-chain acyl-CoAs. Subsequently, we observed that the addition of myristoyl-CoAs and oleoyl-CoAs disrupted the MftR–O_mft_ complex (Fig. 4*A*, lanes 8 and 9) and resulted in both bound and unbound O_mft_. Conversely, the addition of fatty acid carboxylates did not result in the same disruption of the MftR–O_mft_ complex (Fig. 4*A*, lane 10), suggesting that CoA is a requisite for acyl-CoA binding. However, CoA alone did not disrupt the MftR–O_mft_ complex either (Fig. 4*A*, lane 4), suggesting that protein contacts with the ligand rely on both the fatty acid and the CoA.

ITC experiments were performed to validate the competitive EMSA findings and to determine the specificity and affinity of MftR toward acyl-CoAs. A typical thermogram was obtained when oleoyl-CoA was titrated into MftR (Fig. 4*B*). The *K*_*d*_ value (1.4 ± 0.1 μM; Fig. 4*C*) and the number of binding sites (∼0.6 sites per monomer MftR) were obtained from the nonlinear one-site model to the normalized fitting curve. The *K*_*d*_ value is comparable to known mycobacterial TFRs that are activated by oleoyl-CoA (39, 40). To establish the specific acyl-CoA(s) that activate MftR, we measured the *K*_*d*_s for myristoyl-CoA, palmitoyl-CoA, and steroyl-CoA and found the values to be within the 2 to 4 μM range (Fig. 4*C*). Of note, we observed a ∼10-fold decrease in binding affinity with lauroyl-CoA as compared with oleoyl-CoA. This drop in affinity is consistent with EMSAs that indicated medium-chain and short-chain acyl-CoAs do not disrupt the MftR–O_mft_ complex. Taken together with the EMSAs, our ITC data suggest that MftR, and thus likely MFT biosynthesis, is activated by long-chain acyl-CoAs.

### Structural contributions to ligand binding

**Figure 5:**
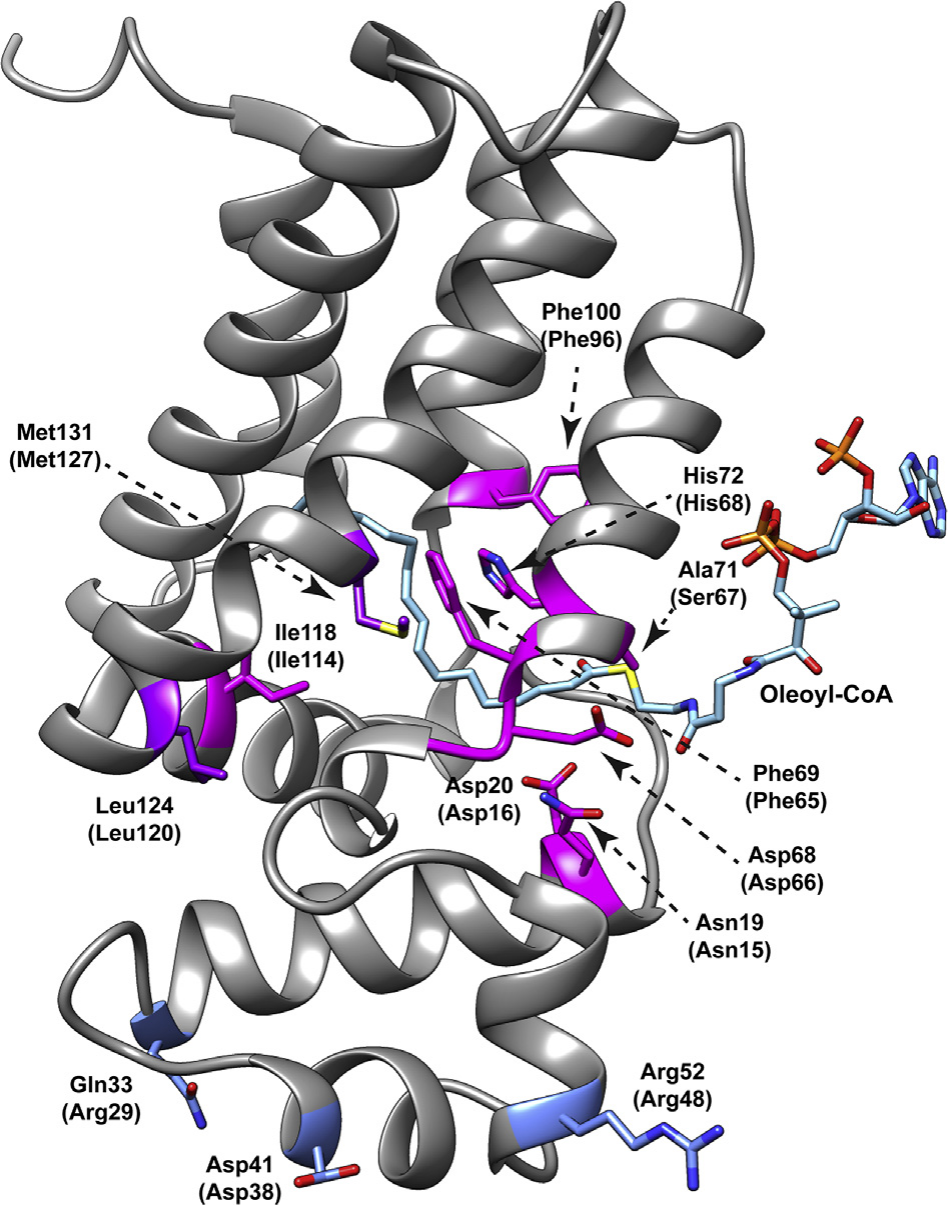
**A docked structure of oleoyl-CoA bound to MftR.** The *Rhodococcus jostii* RHA1 MftR (2RAE, *gray*) was docked with oleoyl-CoA (*blue*) using SwissDock. Shown is the lowest energy model (−10 kJ/mol) calculated. Residues associated with the oleoyl-CoA–binding pocket (*magenta*), DNA-binding motif (*blue*), and at the interface (*purple*) are annotated for both 2RAE and the corresponding *Mycobacterium smegmatis* mc^2^155 MftR (*parenthesis*).

Next, we examined which amino acid residues contribute to the interaction between MftR and oleoyl-CoA. The crystal structure of Msmeg MftR is currently unavailable; however, an unpublished structure of *Rhodococcus jostii* RHA1 MftR has been deposited in the Protein Data Bank (PDB) (PDB ID: 2RAE, 54% identical, Fig. S3). Using this structure, we employed SwissDock to model oleoyl-CoA bound to MftR (Fig. 5) (41, 42). From the docked structure, we identified eight conserved residues on MftR that were expected to create the acyl-binding pocket or bind CoA through electrostatic interactions. Following single amino acid replacements of the residues, we measured the *K*_*d*_ values of the mutant proteins to oleoyl-CoA using ITC. For the putative acyl-binding pocket residues Phe65, Phe96, and Ile114, mutations to alanine led to no or modest change to the *K*_*d*_ for oleoyl-CoA (Table 1). This is consistent with other TFR proteins where single-residue changes to the acyl-binding pocket resulted in little to no change in their *K*_*d*_ to acyl-CoAs (39). However, we cannot rule out the possibility that Phe65, Phe96, and Ile114 do not participate in acyl-CoA binding. Conversely, when His68 was mutated to alanine, binding of oleoyl-CoA by the protein was undetectable. To validate this observation, competitive EMSAs were carried out using the H68A mutant. Here, it was observed that the H68A mutant remained bound to O_mft_ even in the presence of 100 μM oleoyl-CoA (Fig. S4*A*). Thus, it is highly likely that His68 is an important residue for oleoyl-CoA binding. To evaluate residues that were expected to interact with CoA, Gln15, Asp16, Ser67, and Asp66 were targeted. Of these, mutants of Asp16 and Ser67 had the greatest effect (Table 1). For instance, the S67A mutation led to a fivefold increase in the *K*_*d*_ value for oleoyl-CoA and the addition of bulk, by the mutation S67W, led to an eightfold increase in the *K*_*d*_ value. Likewise, the D16W mutant, which increased bulk and removed the negative charge, led to a fivefold increase in the *K*_*d*_ for oleoyl-CoA. Moreover, when the charge was changed from negative to positive by the mutant D16R, binding of oleoyl-CoA was no longer detectable. This latter observation was validated using competitive EMSAs (Fig. S4*B*). Even in the presence of 100 μM oleoyl-CoA, the D16R mutant remained bound to O_mft_. Taken together, it is likely that Ser67 and Asp16 participate in oleoyl-CoA binding, likely through electrostatic interactions.

**Table 1:** Dissociation constants for MftR variants and oleoyl-CoA measured by ITC

MftR variant	*K*_*d*_ (μM)^a^
WT	1.4 ± 0.1
Q15A	2.0 ± 0.1
D16R	ND
D16W	4.8 ± 0.2
F65A	1.9 ± 0.1
D66A	1.6 ± 0.1
S67A	5.4 ± 0.2
S67W	8.3 ± 0.2
H68A	ND
F96A	1.4 ± 0.1
I114A	2.4 ± 0.1
L120A	5.2 ± 0.2
L120R	6.2 ± 0.2
M127A	4.8 ± 0.1

Abbreviation: ND, not detected.

a The *K*_*d*_ values are reported as the average of at least three independent experiments and the standard deviation.

Next, to determine which residues are important for the MftR–DNA interaction, we targeted three residues (Fig. 5; Arg29, Asp38, and Arg48) on the HTH domain for site-directed mutagenesis. Similar residues have been shown to be important for DNA–protein contacts in other TFRs (43, 44, 45). The *K*_*d*_s of the MftR mutants and O_mft_ were measured by FP (Table 2). As expected, the alanine mutants for Arg29 and Arg48 had a significant impact on the ability of MftR to bind O_mft_, with a 10-fold and 20-fold increase in the *K*_*d*_ values, respectively (Table 2). It is possible that Arg29 and Arg48 interact with the phosphate backbone on O_mft_, thus making them important for DNA binding. Interestingly, the D38A mutant abolished the ability of MftR to binding O_mft_ (Table 2). To corroborate this observation, an EMSA was carried out with D38A mutant and O_mft_ (Fig. S4*C*). The addition of up to 100 μM of the D38A mutant to O_mft_ resulted in a single unbound species, confirming that Asp38 is important for binding to O_mft_. The importance of Asp38 is likely explained by Asp38 forming of a salt bridge with Arg47 upon binding DNA. A similar salt bridge has been shown to be important for the master regulator Msmeg_6564 (46). In Msmeg_6564, the hydrogen bond between Glu37 and Lys47 (∼2.5 Å; PDB ID: 4JL3) stabilizes the HTH domain in the major groove of DNA and was shown to be important for DNA interaction (46). Nevertheless, until a DNA-bound structure of MftR becomes available, role of Asp38 in O_mft_ binding remains speculative.

**Table 2:** Dissociation constants for MftR variants and O_mft_ measured by FP

MftR variant	*K*_*d*_ (μM)^a^
WT	1.3 ± 0.6
R29A	20.5 ± 2.0
D38A	ND
R48A	13.0 ± 1.1
L120R	28 ± 6.0
M127A	13 ± 6.0

Abbreviation: ND, not detected.

a The *K*_*d*_ values are reported as the average of at least three independent experiments and the standard deviation.

Finally, we examined the effects of mutations at the interface of the DNA-binding and effector-binding domains. Here, residues Leu120 and Met127 were mutated to arginine and alanine, respectively, and the *K*_*d*_s for oleoyl-CoA and O_mft_ were measured as described previously. While we did not observe significant impact on the *K*_*d*_ for oleoyl-CoA (Table 1), we did find that the L120R and M127A mutants impaired the ability of MftR to bind O_mft_, with an observed increase in *K*_*d*_ values by 10-fold and 25-fold, respectively (Table 2). This suggests that disruption of the packing between the DNA-binding domain and the regulatory domain impacts binding of DNA substantially more than oleoyl-CoA.

### MFT biosynthesis is induced by oleoyl-CoA *in vivo*

To validate that oleoyl-CoA is an effector of MFT *in vivo*, we turned to a well-established far-red reporting system designed to detect gene expression in mycobacteria (47). We repurposed the pCherry3 vector by replacing the existing constitutive promoter, P_smyc_, with *mftR*–P_mft_–*mftA* and in frame with mCherry (Fig. S5) yielding P_mft_–pCherry. As a result, the plasmid encodes *mftR* under its native promoter, the *mft* promoter, and an *mftA–mCherry* gene fusion. The addition of *mftR* is expected to suppress background fluorescence that may arise by having increased copies of P_mft_ and insufficient MftR to repress expression. Transcription and translation of *mftA* is expected to result in a fluorescent MftA–mCherry reporter, the intensity of which can be measured through as a function of time. Together, the P_mft_–pCherry reporter system was expected to provide information about the timing and relative abundance of MftA production.

**Figure 6:**
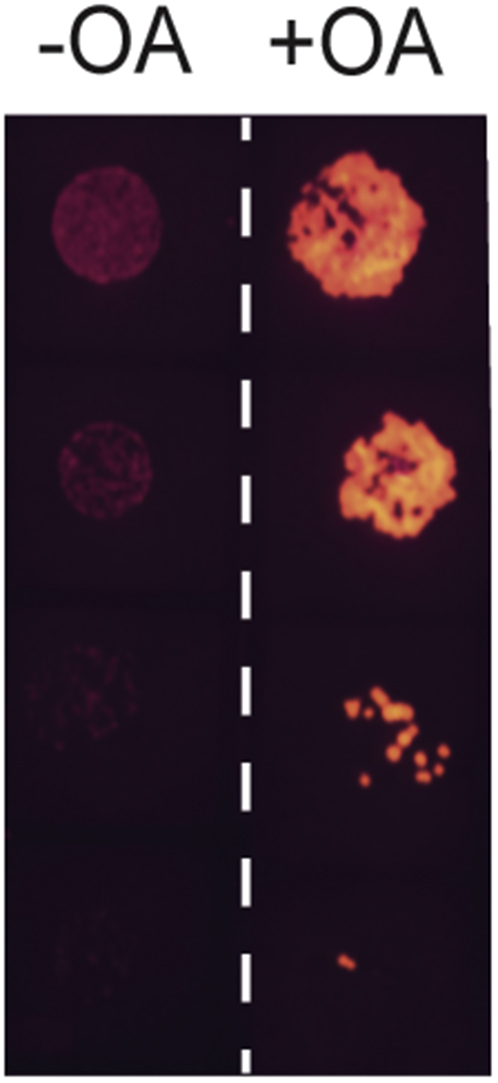
**Induction of MFT biosynthesis by oleoyl-CoA *in vivo*.** A spot test growth analysis of *Mycobacterium smegmatis* mc^2^155 transformed with the plasmid P_mft_–pCherry encoding for the *mftR*, the regulatory region of the *mft* BGC, and a *mftA–mCherry* gene fusion. Approximately 500, 100, 25, and 5 cells were grown on 7H9 supplemented with 0.1% w/v glucose or 0.05% w/v each glucose and oleic acid (OA). Fluorescence images were obtained simultaneously from two different petri dishes using 585 nm excitation and 635 nm emission wavelengths. The *white dashed line* represents the splice point between images. 7H9, Middlebrook 7H9; BGC, biosynthetic gene cluster; MFT, mycofactocin; P_mft_, *mft* promoter.

**Figure 7:**
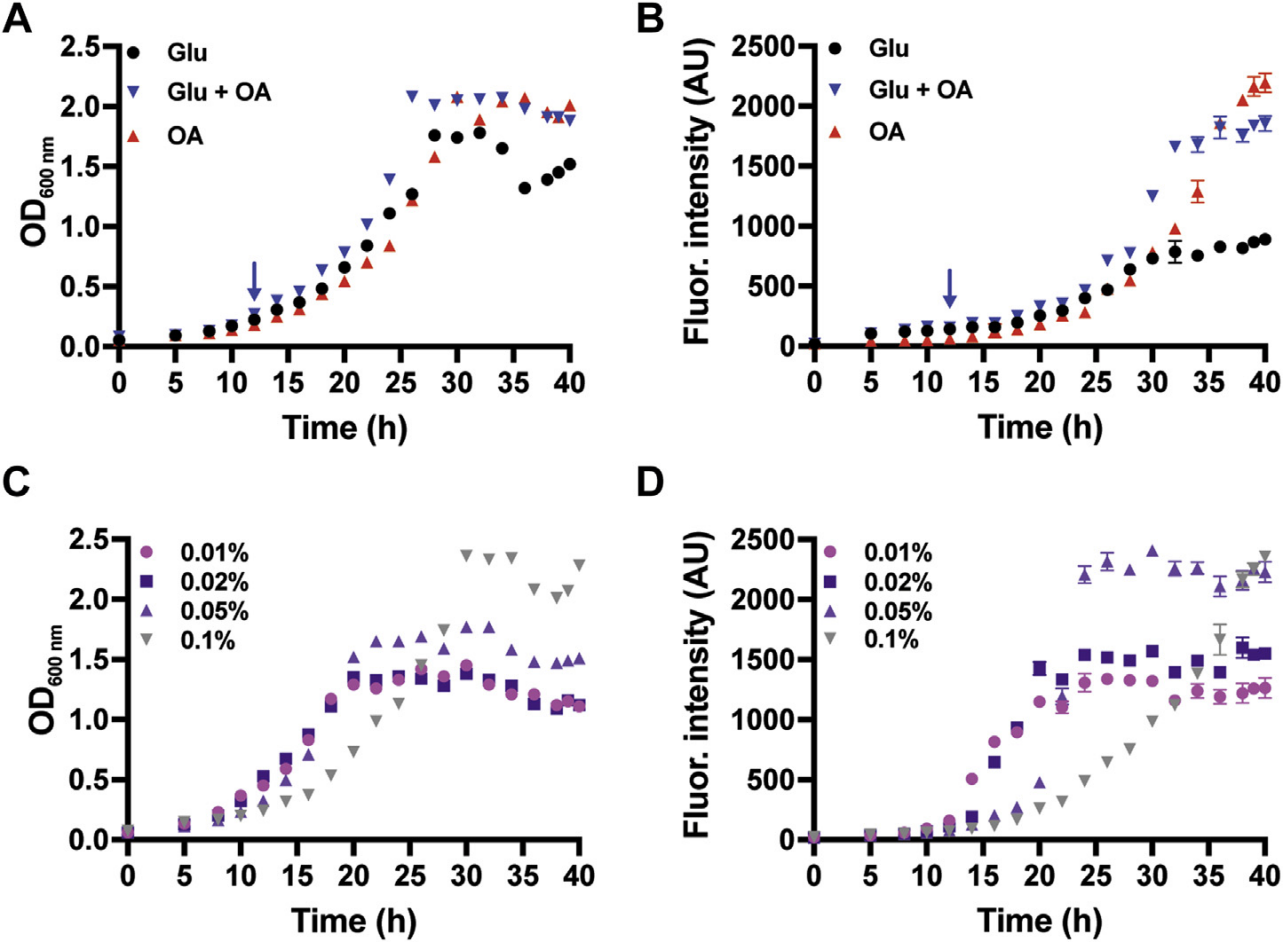
**Growth curve assays with Msmeg/P**_**mft**_**–pCherry.***A*, absorbance at 600 nm measurements for the 7H9 media supplemented with 0.1% tyloxapol, 50 μg/ml hygromycin, and glucose and/or oleic acid (OA) as carbon sources. Concentrations of carbon sources were glucose only (0.1% w/v), OA only (0.1% w/v), and glucose/OA (0.05% w/v each) where OA was added after 12 h (*blue arrow*). *B*, corresponding fluorescence intensities (excitation of 585 nm/emission of 612 nm) of Msmeg/P_mft_–pCherry cultures described in *A*. *C*, absorbance at 600 nm measurements for dose-dependent addition of OA to the 7H9 media supplemented with 0.05% w/v glucose and 0.01, 0.02, 0.05, 0.1% w/v of OA. *D*, corresponding fluorescence intensities of Msmeg/P_mft_–pCherry cultures described in *C*. 7H9, Middlebrook 7H9; Msmeg, *Mycobacterium smegmatis* mc^2^155; P_mft_, *mft* promoter.

To directly establish that oleoyl-CoA induces MFT biosynthesis *in vivo*, a colony dilution experiment was carried out using Msmeg transformed with P_mft_–pCherry grown on Middlebrook 7H9 (7H9) supplemented with glucose or oleic acid (OA). We used OA in the growth media since it is well known that bacterial fatty acid shuttle systems convert free fatty acids to the corresponding acyl-CoAs and since Msmeg encodes for at least two known fatty acid transporters (48, 49). As shown in Figure 6, the fluorescence intensity of Msmeg colonies containing the reporter system is starkly increased in the presence of OA as compared with glucose alone. To assess when this occurs in real time, growth curve assays with Msmeg/P_mft–_pCherry were carried out. When cell cultures were grown to nearly the same absorbance (Fig. 7*A*), the fluorescence intensity of Msmeg/P_mft_–pCherry supplemented with 0.1% OA only is nearly three times in magnitude as compared with the control (Fig. 7*B*). We observed the same effect when OA was added to the media after 12 h of incubation. Next, we determined if MFT induction is dependent on the concentration of OA. We carried out a titration series of growth curves where 7H9 media were supplemented with 0.05% w/v glucose and 0.01, 0.02, 0.05, or 0.1% w/v OA. As shown in Figure 7, *C* and *D*, the addition of 0.05% w/v OA produced the highest fluorescence intensity within a time frame of 40 h. However, in general, increasing concentrations of OA had little effect on the overall production of the MftA–mCherry fusion. The lack of immediate production of MftA–mCherry after the addition of OA and the concentration independence is perplexing. Since we did not observe a diauxic growth curve, we do not suspect that Msmeg is displaying a prototypical substrate bias. Rather, we expect that a subtler and unknown mechanism is at play.

## Discussion

The *mft* BGC is one of the most widely distributed RiPP biosynthetic pathways and is highly concentrated in mycobacteria genomes. Of the ∼625 unique species that encode for MFT biosynthesis, 300 are found in the mycobacteria genus (8) including many pathogenic mycobacteria species. Despite the wide distribution of the *mft* BGC and its frequent occurrence in mycobacterial pathogens, little is known about the physiological conditions that lead to MFT production. To address this gap in knowledge, we focused our efforts on the putative regulator MftR, which has remained relatively unexplored. As a point of clarification, it should be noted that the gene designation of MftR used throughout this article is derived from automated gene annotation used by UniProt and the National Center for Biotechnology and Information and should not be confused with the MarR-family regulatory protein that has been named *m*ajor *f*acilitator *t*ransport *r*egulator (UniProt: Q2SVY7) and is associated with urate response in *Burkholderia* sp. (50, 51).

In this study, we demonstrated that MftR binds to the promoter region of the *mft* gene cluster, suggesting that it acts as a *cis* regulatory element in the transcription of the *mft* BGC. Notably, we used DNase I footprinting to sequence the DNA-binding region of MftR and identified a stretch of 27 bp residing −79 bp upstream to the start of the *mft* operon. We confirmed that MftR binds the 27 bp region by measuring the *K*_*d*_ of the MftR–DNA complex by fluorescence anisotropy. In addition, we used qRT–PCR and growth assays to demonstrate that overexpression of *mftR* reduces transcript levels of the *mft* BGC. In addition, we found that the *mft* operator is conserved in both sequence and location in the other mycobacterial genomes. This suggests that MftR homologs likely regulate the *mft* BGC using a similar operator sequence. Our findings are consistent with pathogen-sequencing data from *in vivo* samples of macrophages infected with MTB. In this study, Peterson *et al.* (36) found that Rv0691c is highly expressed and affects nearly 50 gene targets. Importantly, they found that upregulation of Rv0691c resulted in the repression of *mftB*, *mftC*, and *mftD* transcription. In addition, our findings are consistent with regulatory networks that were built for transcription factors in MTB (21). Using chromatin immunoprecipitation sequencing experiments, Minch *et al.* (21) found that Rv0691c binds to a region 5′ to the *mftA* homolog Rv0691a. The motif consensus described by Minch *et al.* is conserved in the O_mft_ sequence, which is shown in Figure 2*E*. Taken together with our sequencing, binding affinity data, qRT–PCR data, and bioinformatics data, it is logical to conclude that MftR is an *mft* BGC transcriptional repressor in mycobacteria that harbor both the *mft* BGC and *mftR*.

Significantly, we found that MftR is activated by long-chain acyl-CoAs. Using competitive EMSAs and ITC, we showed that acyl-CoAs ranging from C12 to C18 are effectors of MftR *in vitro*. In addition, we repurposed a fluorescent reporter system to show that the *mft* BGC is upregulated in Msmeg cultures when oleate is supplemented to growth media as compared with glucose alone. These findings suggest that MFT production and utilization is required for some aspect of fatty acid metabolism. Both bioinformatics and direct evidence support this view. In Msmeg, putative MFT-dependent dehydrogenases belonging to the TIGR03989 family are colocalized with fatty acid–modifying enzymes. For instance, *msmeg_4801* is colocalized with a putative 3-oxo-acyl carrier protein reductase, and *msmeg_2204* is in a gene cluster that contains an acyl-CoA dehydrogenase. More directly, *in vivo* studies have shown that the *mymA* operon, consisting of the genes *rv3083–rv3089*, is required for cell wall maintenance (52, 53) and for maintaining the mycolic acid composition in MTB when exposed to acidic pH (54). Encoded in the *mymA* operon is Rv3086, a putative MFT-dependent dehydrogenase. While enzymatic activity has yet to be established for Rv3086, it has been proposed to carry out the conversion of terminal methyl groups of fatty acids to carboxylic groups for condensation (53). Therefore, it is likely that the activation of the *mft* BGC by acyl-CoAs is due to MFT-dependent dehydrogenases that are associated with fatty acid metabolism.

We have found that MftR is at least one regulator of MFT biosynthesis. While our findings significantly progress current knowledge about the physiological conditions that induce MFT biosynthesis, we recognize that the regulatory network of MFT biosynthesis is likely incomplete. For instance, it is known that the MFT-dependent dehydrogenases Msmeg_6242 and Msmeg_1410 are required for primary alcohol and carveol catabolism, respectfully (13, 18); yet neither catabolic pathway incorporates long-chain acyl-CoAs. Therefore, we expect that other regulators influence the timing of MFT production. Supporting this notion, the MTB regulator, Rv0678, controls the expression level of *mmpS5–mmpL5* genes, which are associated with azole efflux (55). In addition, chromatin immunoprecipitation sequencing experiments show that Rv0678 also binds to regions of DNA that encode for MftB and MftD, potentially making Rv0678 a secondary regulator of MFT biosynthesis in MTB (21). While the regulatory network for MFT biosynthesis may not be complete, our findings here provide the new insight to the physiological conditions that lead to MFT production.

Despite the rapid progress in solving the biosynthesis, structure, and function of MFT, little is known about the physiological processes that require the molecule. Previously, we speculated that MftR is a regulator of MFT biosynthesis (8). The present study confirms this to be true, at least in Msmeg. In summary, we provide here mechanistic insights into the MftR-dependent regulation of MFT biosynthesis and demonstrate that the induction of MFT biosynthesis is acyl-CoA dependent. Overall, this study underpins the importance of MFT in mycobacteria. However, to fully grasp the physiological roles that MFT participates in, future studies should be dedicated to investigating chemistries of MFT-dependent dehydrogenases.

## Experimental procedures

### Materials

All acyl-CoAs were purchased from Sigma–Aldrich. Oligonucleotides used in this study were synthesized by Invitrogen and are listed in Table S1. Double-stranded O_mft_, and mutants thereof, were prepared from single-stranded oligonucleotides by mixing two oligos in equal molar amounts, heating the mixed oligos for 5 min at 95 °C and then gradually cooling the oligos to the room temperature. DNase I footprinting assays were contracted to Profacgen.

### Expression and purification of MftR

The MftR gene (UniProt: A0QSB5) from *M. smegmatis* mc^2^155 was cloned into pET-28a (Novagen) using the NdeI and XhoI restriction sites. The sequence-verified plasmid was transformed into *E. coli* BL21(DE3) pLysS cells. An overnight culture was used to inoculate 1 l of terrific broth. The cells were grown at 37 °C and 220 rpm until an absorbance reached ∼1.0 at 600 nm and at which point 1 mM IPTG was added to induce protein production. The temperature was dropped to 21 °C, and the cells were grown overnight. The cells were then centrifuged at 5000*g* for 10 min at 4 °C. The resulting pellet was resuspended in five times volume of lysis buffer (50 mM Hepes, 300 mM NaCl, 40 mM imidazole, and pH 7.5). To the suspension, 1% w/v 3-[(3-cholamidopropyl)dimethylammonio]-1-propanesulfonate, 0.1 mg/g of lysozyme, and 0.05 mg/g of DNase were added, and the lysate was stirred for 20 min at room temperature. The lysate was disrupted by sonication at 50% output with a pulse of 3 s on and 3 s off for 5 min. The lysate was centrifuged at 20,000*g* for 10 min, and the supernatant was loaded onto a 5 ml His-Trap column (GE Healthcare) using Akta Pure FPLC. The bound protein was washed with 25 ml lysis buffer, eluted with elution buffer (50 mM Hepes, 300 mM NaCl, 300 mM imidazole, and pH 7.5), and buffer exchanged into storage buffer (50 mM Hepes, 300 mM NaCl, 10% glycerol, and pH 7.5) over a HiPrep 26/10 desalting column. The protein was concentrated using 10 kDa spin concentrator (Millipore) at 5000*g* for 20 min at 22 °C. The purity of the protein was evaluated by SDS-PAGE.

### Construction of P_mft_–*mftA*–pCherry

The *mft* promoter region from *M. smegmatis* mc^2^155 was synthesized and cloned into the pCherry vector (47) by Genscript (see Supporting Information for the full sequence). In short, the native sequence for the *mft* promotor region from ×698 to 0 nt through the native sequence of *mftA* (+93 nt) was cloned into the XbaI and BamHI restriction sites of pCherry, replacing the existing *smyc* promoter. The existing pCherry fluorescent protein was fused to the C terminus of MftA by removing the native stop codon in the *mftA* sequence. The full sequence of the plasmid can be found in the Supporting Information.

### Construction of pMftR+

The wildtype *mftR* gene from *M. smegmatis* was PCR amplified from genomic DNA and cloned into the pCherry vector (47) using the BamHI and HindIII restriction sites.

### EMSAs

His-tagged MftR was used to assess the protein binding to P_mft_–MftA promoter fragment. DNA was mixed with increasing concentrations of the MftR protein in the reaction buffer (50 mM Hepes, 50 mM NaCl 0.1 mM EDTA, and pH 7.5) and incubated at room temperature for 10 min. The reactions were resolved by electrophoresis on a 5% (v/v) nondenaturing polyacrylamide gel in 1× Tris/borate/EDTA buffer at 200 V for 20 min on ice. Prior to the analysis, the gel was prerun for 30 min at 150 V on ice. Results were visualized by GelRed and recorded using Azure Biosystems 600 imaging system.

### Preparation of fluorescent FAM-labeled probes

The promoter region was PCR amplified with 2× TOLO HIFI DNA polymerase premix from P_mft_–pCherry. The FAM-labeled probes were purified by the Wizard SV Gel and PCR Clean-Up System (Promega) and quantified with NanoDrop 2000C (Thermo Fisher Scientific).

### DNase I footprinting assays

DNase I footprinting assays were performed similar to the study by Wang *et al.* (56). Approximately, 350 ng of DNA probes were incubated with 0 and 2 μg of MftR in a total volume of 40 μl. After incubation for 30 min at 30 °C, a 10 μl solution containing approximately 0.015 U DNase I (Promega) and 100 nmol freshly prepared CaCl_2_ was added to the probe/MftR mixture and further incubated at 37 °C for 1 min. The reaction was stopped by adding 140 μl DNase I stop solution (200 mM unbuffered sodium acetate, 30 mM EDTA, and 0.15% SDS). Samples were first extracted with phenol/chloroform and then precipitated with ethanol. Pellets were dissolved in 30 μl MilliQ water. The preparation of the DNA ladder, electrophoresis, and data analysis were as described in the study by Wang *et al.*, except that the GeneScan-LIZ600 size standard (Applied Biosystems) was used.

### FP assays

FP-binding assays were carried out in binding buffer (50 mM Hepes, 50 mM NaCl, and pH 7.5) containing 0.5 μM fluorescein-labeled O_mft_ using Tecan Infinite M1000. The MftR protein was titrated into the binding solution. The fluorescein-labeled O_mft_ was mixed with increasing concentrations of MftR in the 96-well plate (Corning), similar to the EMSA reactions, and were incubated for 10 min before the measurements were taken. Excitation and emission wavelengths of 470 and 525 nm were monitored. All the experiments were done in triplicate. The G-factor was calculated from a solution of free fluorophore. The data were plotted and analyzed using GraphPad Prism (GraphPad Software, Inc). The mutated fluorescein-labeled O_mft_ was measured as described previously.

### Construction of MftR mutants

Mutant strand synthesis reactions were performed according to the Agilent QuikChange site-directed mutagenesis protocol. The *mftR*_pET28a plasmid was used as a template for site-directed mutagenesis studies. The forward primers used for the single-point mutations are shown in Table S1. Sequence-verified plasmids were transformed into *E. coli* BL21(DE3) pLysS cells for protein production and purified using the same protocol as wildtype MftR.

### ITC measurements

ITC was performed using TA Nano ITC. MftR was loaded in the sample cell at the concentration of 16 and 250 μM acyl-CoAs (C12–C18) loaded in the syringe. To minimize the effect of buffer mismatch, the stock concentration of acyl-CoAs was prepared using ITC buffer (50 mM Hepes, 50 mM NaCl, and pH 7.5), and the pH was adjusted to within 0.05 units. In addition, MftR was buffer exchanged into ITC buffer using a PD-10 column (GE Life Sciences). The volume of the titrant added at each injection into the sample cell was 2.22 μl. The time interval between the successive injections was 180 s. The temperature of the cell was kept at 25 °C. The data obtained were fit by independent one-site model using NanoAnalyze Data Analysis, version 3.8.0, that was provided with the instrument.

### Electroporation of *M. smegmatis*

Electrocompetent *M. smegmatis* mc^2^155 cells were prepared as described (57). In short, 1 l of 7H9–OA–albumin–dextrose–catalase media was inoculated with Msmeg and incubated at 37 °C with shaking until an absorbance reached ∼1.0 at 600 nm. The cell culture was incubated on ice for 1.5 h, and the cells were harvested by centrifugation at 2000*g* for 10 min. The pelleted cells were suspended in 500 ml ice-cold 10% glycerol and centrifuged again. Following two additional wash processes, the cells were suspended in 25 ml of ice-cold 10% glycerol and transferred to a 50 ml conical tube. The cells were pelleted by centrifugation as described previously, suspended in 4 ml of ice-cold 10% glycerol, aliquoted to 300 μl, and flash frozen or used immediately. To a thawed aliquot of competent cells, ∼1 μg of plasmid DNA was added and incubated on ice for 5 min. The cells were transferred to a 0.2 cm electrode gap cuvette and pulsed with a Gene Pulser (Bio-Rad) set to 2.50 kV, 25 μF, and 1000 Ω. Following electroporation, 1 ml of 7H9–OA–albumin–dextrose–catalase was added to the cells. The suspension was then incubated for 2 h at 37 °C and plated on 7H10–ADC plates supplemented with 50 μg/ml of hygromycin B.

### Growth curve analysis

For fluorescence-based assays, a single Msmeg colony harboring P_mft_–pCherry was used to inoculate 25 ml of 7H9 media supplemented with 0.1% tyloxapol, 0.1% w/v glycerol, 50 μg/ml hygromycin, and 0.1% w/v glucose. For cultures containing OA only, 0.1% w/v OA was used. For cultures containing both OA and glucose, 0.05% w/v glucose was used, and OA was added 12 h after to the concentration of 0.05% w/v. For dose-dependent cultures containing OA (0.01%, 0.02%, 0.05%, 0.1% w/v), glucose was supplemented to the media to 0.05% w/v. The starter culture was incubated at 37 °C with shaking overnight. The overnight culture was used to inoculate 7H9 media to an absorbance of 0.05 at 600 nm (1 cm pathlength) with carbon sources supplemented as described previously. The cultures (30 ml) were incubated at 37 °C with shaking for 40 h. Aliquots were taken every 2 h, and absorbance at 600 nm values were measured. To track the expression of the P_mft_–pCherry fusion, fluorescence measurements were taken on a Cary Fluorimeter every 2 h using an excitation of 585 nm and an emission of 612 nm.

### Spot plate dilution growth analysis

A single time point growth assay was standardized for the growth of Msmeg on 7H9 agar plates with various carbon sources supplemented. Briefly, a primary culture of Msmeg/P_mft_–pCherry was grown in 7H9 supplemented with 0.1% w/v glucose overnight. The absorbance was measured and converted to colony-forming units (CFUs) using the standard 3.13 × 10^7^ CFU·ml^−1^·absorbance^−1^ conversion factor (58). Five-fold serial dilutions of cultures were made in 7H9 base media to yield 500, 100, 25, and 5 CFU/μl solutions. Two microliters of each dilution were transferred to 7H9 plates containing 0.1% w/v carbon source, and the spots were allowed to dry. Following incubation at 37 °C for 3 days for the growth of individual colonies, plates were imaged using an Azure 600 fluorescent imager under identical conditions.

### RNA isolation, complementary DNA synthesis, and qPCR

RNA was purified from *M. smegmatis* using a Zymo Fungal/Bacterial RNA MiniPrep Kit, and contaminating DNA was removed using a Zymo RNA Clean and Concentrator Kit. The quality of RNA was checked by agarose electrophoresis prior to subsequent steps. Complementary DNA was synthesized from RNA samples using the First Strand cDNA Synthesis Kit from NEB. qPCRs were performed using Luna Universal qPCR Master Mix (NEB) following the recommended protocols. Primer pairs were designed using the IDT Primer Quest Tool and were evaluated according to the standard curve method. mRNA expression data were normalized to SigA. The primer sequences and efficiencies are listed in Table S1.

## Data availability

All data are contained within the article.

## Supporting information

This article contains supporting information.

## Conflict of interest

The authors declare that they have no conflict of interest with the contents of this article.
