# Supplementary material for: Biosynthesis of the redox cofactor mycofactocin is controlled by the transcriptional regulator MftR and induced by long-chain acyl-CoA species
Source: J Biol Chem. 2021 Dec 9;298(1):101474. doi: 10.1016/j.jbc.2021.101474 (PMC8728441; doi:10.1016/j.jbc.2021.101474)
Supplement: Supplemental Figures S1–S5 and Table S1 [file mmc1.docx]

**Supporting Information**

**Biosynthesis of the redox cofactor mycofactocin is controlled by the transcriptional regulator MftR and induced by long chain acyl-CoA species.**

Aigera Mendauletova^1^ and John A. Latham^1,*^

1. Department of Chemistry and Biochemistry, University of Denver, Denver, Colorado 80210, USA

*Corresponding author: [john.latham@du.edu](mailto:john.latham@du.edu)

Contents:

Figure S1. SDS-PAGE gel of WT MftR.

Figure S2. EMSA controls showing the specificity of MftR towards the O_mft_.

Figure S3. A sequence alignment between 2RAE and Msmeg MftR.

Figure S4. Validation of MftR mutants for oleoyl-CoA and DNA binding sites using EMSA.

Figure S5. A schematic representation of the engineered plasmid encoding for the promoter region of *mft* and *mftA-mCherry* gene fusion.

Table S1. Primers used in the study.

DNA Sequences

**Figure S1 –** Sodium dodecyl sulfate-polyacrylamide gel electrophoresis analysis of WT MftR. The purified protein (4 μg) was analyzed on 15% polyacrylamide gel, stained with Coomassie Brilliant Blue G-250. Lane 1 is the blue stain protein marker.


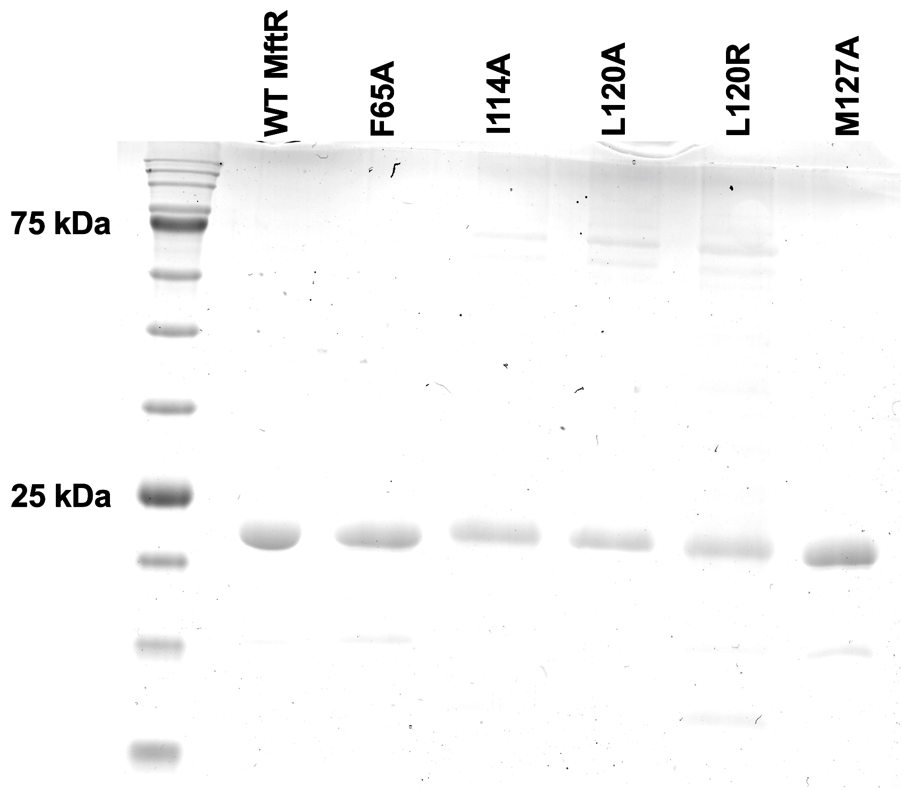


**Figure S2 –** O_mft_ control with “cold” competitive ligand and a non-specific DNA sequence. Unlabeled “cold” probe (4μM) was mixed with WT MftR (2μM) left at room temperature to react for 30 min. FAM-labeled O_mft_ (20 nM) was then added to the reaction and left for additional 30 minutes (lane 3). Unlabeled non-specific DNA sequence (4μM) was mixed WT MftR (2μM) left at room temperature to react for 30 min. FAM-labeled O_mft_ (20 nM) was added and left to react for additional 30 minutes (lane 4). Protein-DNA complex was separated by electrophoresis on 5% polyacrylamide gel and imaged using FAM excitation and emission wavelengths. The U and B represent unbound and bound fractions respectively.


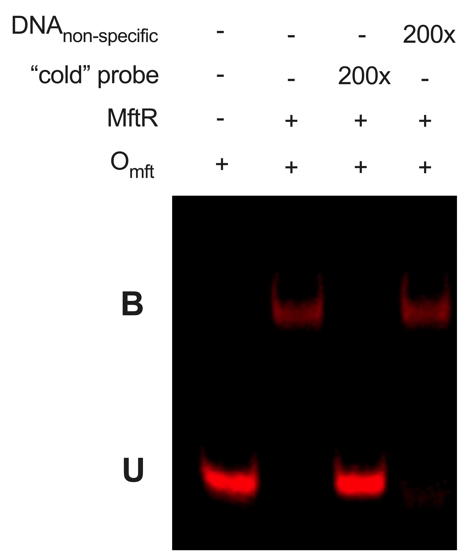


**Figure S3** A sequence alignment between 2RAE and Msmeg MftR.

**Figure S4 –** EMSA validating the importance of MftR_H68A_, MftR_D16R_, and MftR_D38A_ mutants. **A)** FAM-labeled O_mft_ (100nM) was mixed with MftR_H68A_ (2μM) and left to react for 10 min at room temperature. Increased concentrations of oleoyl-CoA were added into the reactions (1, 10, 50, 100 μM). The U and B represent unbound and bound fractions respectively. Protein-DNA complex was separated by electrophoresis on 5% polyacrylamide gel and imaged using FAM excitation and emission wavelengths. **B)** FAM-labeled O_mft_ (100nM) was mixed with MftR_D16R_ (2μM) and left to react for 10 min at room temperature. Increased concentrations of oleoyl-CoA were added into the reactions (1, 10, 50, 100 μM). **C)** FAM-labeled O_mft_ (100nM) was mixed with increased concentrations of MftR_D38A_ (up to 100μM) and left to react for 20 min at room temperature. Protein-DNA complex was separated by electrophoresis on 5% polyacrylamide gel and imaged using FAM excitation and emission wavelengths.


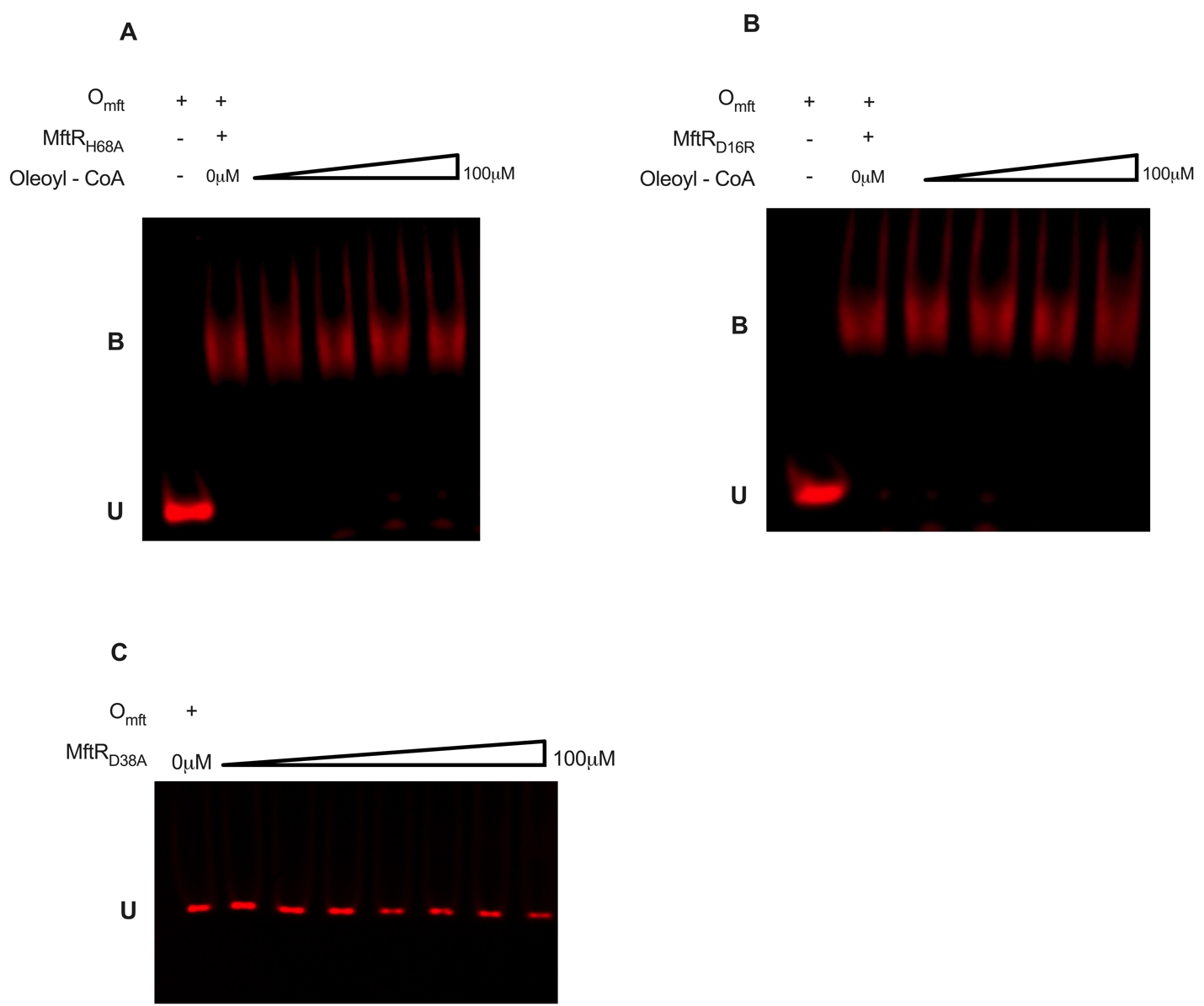


**Figure S5** – A schematic representation of the engineered plasmid encoding for the promoter region of *mft* and *mftA-mCherry* gene fusion. The pCherry 3 vector was remodeled by replacing P_smyc_ with P_mft_*-mftA* and in frame with mCherry.


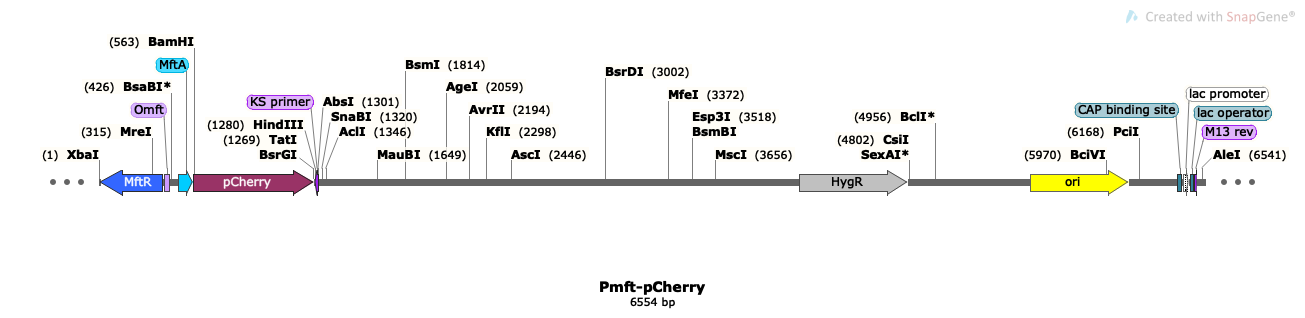


**TABLE S1**. Primers used in this study

| Purpose | Primer | Sequence $(5'-3')$ |
| --- | --- | --- |
| EMSAs;  FP | FAM-O_mft_ | TCCATTCTGGCACTCGATGCCATATAT |
|  |  |  |
| MftR mutants, ITC | Q15A  D16R  D16W  R29A  D38A  R48A  F65A  D66A  S67A  S67W  H68A  F96A | CGCCGCTCCACCACGGCGGACCACATCGCCGGC  TCCACCACGCAGCGACACATCGCCGGC  TCCACCACGCAGTGGCACATCGCCGGC  GATCTGTTCGCGGCCGCGGGGTTCGACGCGGTC  GCGGTCAGCGTCGACGCGGTCGCGGCCGCCGCG  GCGGGCATCTCGCGAGCGACCCTGTTCCGGTAC  GCAGGTGTGAGTCGGCATCACCCCACGGGATG  CCGTGGGGTGATTTCGCGTCACACCTGCAGCAC  TGGGGTGATTTCGACGCGCACCTGCAGCACCTG  GGTGATTTCGACTGGCACCTGCAGCAC  GGTGATTTCGACTCAGCGCTGCAGCACCTGCAG  GAGGCACTGCTGACCGCGAACACCTACGCCGAC |
|  | I114A | GCAGCGTATGCGCGTGGCCCTGGAAACCGAAGA |
|  | L120A | CCTGGAAACCGAAGAGGCGCAGGCGTATTCGATG |
|  | L120R | CTGGAAACCGAAGAGCGGCAGGCGTATTCGATG |
|  | M127A | GCGTATTCGATGACCGCGTACGCCGGCTGGCG |
|  |  |  |

DNA sequences

**Pmft Sequence**

TACGCCTGCAGCTCTTCGGTTTCCAGGATCACGCGCATACGCTGCCGGTGCTCGGCCATCTCGTGGTCGGCGTAGGTGTTGAAGGTCAGCAGTGCCTCGCGCAGGGCGTCGCCGAGTGACACCTCGGAGCTGAGGGTCCTCAGCAGGTTCTGCAGGTGCTGCAGGTGTGAGTCGAAATCACCCCACGGGATGGCGCTCTTGGAGGCGTAGTACCGGAACAGGGTCCGTCGCGAGATGCCCGCGGCGGCCGCGACGTCGTCGACGCTGACCGCGTCGAACCCTCGGGCCGCGAACAGATCGATCGCCACGCCGGCGATGTGGTCCTGCGTGGTGGAGCGGCGACGCCCCGCGCGGGAACCCTCGGACATCTCTCACACCCCCTCTTCCATTCTGGCACTCGATGCCATATATTTGCGATCTCGATCACAACTGTCGAGACCATACGCGACGAAAGGGAGTCCACATGGAACCGAATCAGCACGTCGAGGCCGAGACCGAACTCGTCACCGAGACTCTCGTGGAAGAGGTCTCGATCGACGGTATGTGCGGGGTCTAC

**Pmft-pCherry**

TCTAGATACGCCTGCAGCTCTTCGGTTTCCAGGATCACGCGCATACGCTGCCGGTGCTCGGCCATCTCGTGGTCGGCGTAGGTGTTGAAGGTCAGCAGTGCCTCGCGCAGGGCGTCGCCGAGTGACACCTCGGAGCTGAGGGTCCTCAGCAGGTTCTGCAGGTGCTGCAGGTGTGAGTCGAAATCACCCCACGGGATGGCGCTCTTGGAGGCGTAGTACCGGAACAGGGTCCGTCGCGAGATGCCCGCGGCGGCCGCGACGTCGTCGACGCTGACCGCGTCGAACCCTCGGGCCGCGAACAGATCGATCGCCACGCCGGCGATGTGGTCCTGCGTGGTGGAGCGGCGACGCCCCGCGCGGGAACCCTCGGACATCTCTCACACCCCCTCTTCCATTCTGGCACTCGATGCCATATATTTGCGATCTCGATCACAACTGTCGAGACCATACGCGACGAAAGGGAGTCCACATGGAACCGAATCAGCACGTCGAGGCCGAGACCGAACTCGTCACCGAGACTCTCGTGGAAGAGGTCTCGATCGACGGTATGTGCGGGGTCTACGGATCCATGGTCTCGAAGGGCGAGGAGGACAACATGGCGATCATCAAGGAGTTCATGCGCTTCAAGGTCCACATGGAGGGCTCGGTCAACGGCCACGAGTTCGAGATCGAGGGCGAGGGCGAGGGCCGCCCGTACGAGGGCACCCAGACCGCCAAGCTGAAGGTCACCAAGGGCGGCCCGCTGCCGTTCGCCTGGGACATCCTGTCGCCGCAGTTCATGTACGGCAGCAAGGCCTACGTCAAGCACCCGGCCGACATCCCGGACTACCTGAAGCTGTCGTTCCCGGAGGGCTTCAAGTGGGAGCGCGTCATGAACTTCGAGGACGGCGGCGTCGTCACCGTCACCCAGGACTCGTCGCTGCAGGACGGCGAGTTCATCTACAAGGTCAAGCTGCGGGGCACCAACTTCCCGTCGGACGGCCCGGTCATGCAGAAGAAGACCATGGGCTGGGAGGCCTCGTCGGAGCGCATGTACCCGGAGGACGGCGCCCTGAAGGGCGAGATCAAGCAGCGGCTGAAGCTGAAGGACGGCGGCCACTACGACGCCGAGGTCAAGACCACCTACAAGGCCAAGAAGCCGGTCCAGCTGCCGGGCGCCTACAACGTGAACATCAAGCTGGACATCACCAGCCACAACGAGGACTACACCATCGTCGAGCAGTACGAGCGCGCCGAGGGCCGCCACAGCACCGGCGGCATGGACGAGCTGTACAAGTGAaagcttatcgataccgtcgacctcgagggggggcccggtacgtacccggggatcatcgagccgagaacgttatcgaagttggtcatgtgtaatcccctcgtttgaactttggattaagcgtagatacacccttggacaagccagttggattcggagacaagcaaattcagccttaaaaagggcgaggcctgcggtggtggaacaccgcagggcctctaaccgctcgacgcgctgcaccaaccagcccgcgaacggctggcagccagcgtaaggcgcggctcatcgggcggcgttcgccacgatgtcctgcacttcgagccaagcctcgaacacctgctggtgtgcacgactcacccggttgttgacaccgcgcgcggccgtgcgggctcggtggggcggctgtgtcgcccttgccagcgtgagtagcgcgtacctcacctcgcccaacaggtcgcacacagccgattcgtacgccataaagccaggtgagcccaccagctccgtaagttcgggcgctgtgtggctcgtacccgcgcattcaggcggcagggggtctaacgggtctaaggcggcgtgtacggccgccacagcggctctcagcggcccggaaacgtcctcgaaacgacgcatgtgttcctcctggttggtacaggtggttgggggtgctcggctgtcgctggtgttccaccaccagggctcgacgggagagcgggggagtgtgcagttgtggggtggcccctcagcgaaatatctgacttggagctcgtgtcggaccatacaccggtgattaatcgtggtctactaccaagcgtgagccacgtcgccgacgaatttgagcagctctggctgccgtactggccgctggcaagcgacgatctgctcgaggggatctaccgccaaagccgcgcgtcggccctaggccgccggtacatcgaggcgaacccaacagcgctggcaaacctgctggtcgtggacgtagaccatccagacgcagcgctccgagcgctcagcgcccgggggtcccatccgctgcccaacgcgatcgtgggcaatcgcgccaacggccacgcacacgcagtgtgggcactcaacgcccctgttccacgcaccgaatacgcgcggcgtaagccgctcgcatacatggcggcgtgcgccgaaggccttcggcgcgccgtcgacggcgaccgcagttactcaggcctcatgaccaaaaaccccggccacatcgcctgggaaacggaatggctccactcagatctctacacactcagccacatcgaggccgagctcggcgcgaacatgccaccgccgcgctggcgtcagcagaccacgtacaaagcggctccgacgccgctagggcggaattgcgcactgttcgattccgtcaggttgtgggcctatcgtcccgccctcatgcggatctacctgccgacccggaacgtggacggactcggccgcgcgatctatgccgagtgccacgcgcgaaacgccgaattcccgtgcaacgacgtgtgtcccggaccgctaccggacagcgaggtccgcgccatcgccaacagcatttggcgttggatcacaaccaagtcgcgcatttgggcggacgggatcgtggtctacgaggccacactcagtgcgcgccagtcggccatctcgcggaagggagcagcgcgcacggcggcgagcacagttgcgcggcgcgcaaagtccgcgtcagccatggaggcattgctatgagcgacggctacagcgacggctacagcgacggctacaaccggcagccgactgtccgcaaaaagcggcgcgtgaccgccgccgaaggcgctcgaatcaccggactatccgaacgccacgtcgtccggctcgtggcgcaggaacgcagcgagtggctcgccgagcaggctgcacgccgcgaacgcatccgcgcctatcacgacgacgagggccactcttggccgcaaacggccaaacatttcgggctgcatctggacaccgttaagcgactcggctatcgggcgaggaaagagcgtgcggcagaacaggaagcggctcaaaaggcccacaacgaagccgacaatccaccgctgttctaacgcaattggggagcgggtgtcgcgggggttccgtggggggttccgttgcaacgggtcggacaggtaaaagtcctggtagacgctagttttctggtttgggccatgcctgtctcgttgcgtgtttcgttgcgcccgttttgaataccagccagacgagacggggttctacgaatcttggtcgataccaagccatttccgctgaatatcggggagctcaccgccagaatcggtggttgtggtgatgtacgtggcgaactccgttgtagtgcctgtggtggcatccgtggccactctcgttgcacggttcgttgtgccgttacaggccccgttgacagctcaccgaacgtagttaaaacatgctggtcaaactaggtttaccaacgatacgagtcagctcatctagggccagttctaggcgttgttcgttgcgcggttcgttgcgcatgtttcgtgtggttgctagatggctccgcaaccacacgcttcgaggttgagtgcttccagcacgggcgcgatccagaagaacttcgtcgtgcgactgtcctcgttgatccttgccgagctgggatggaagctcggccgaccaccctggaggagatgatcgaggatgccagggcctttcacgcccgccgctgctgagcgtccgccgccgggcccgcaccgccgtcggccggcccgctccgggctcgcagcagcgggcttcggcgcgggcccggggctcccgagcgcgggcggggctccgggcggccgccgggggccgggggcggcgccgggcggcccggggcgtcaggcgccgggggcggtgtccggcggcccccagaggaactgcgccagttcctccggatcggtgaagccggagagatccagcggggtctcctcgaacacctcgaagtcgtgcaggaaggtgaaggcgagcagttcgcgggcgaagtcctcggtccgcttccactgcgccccgtcgagcagcgcggccaggatctcgcggtcgccccggaaggcgttgagatgcagttgcaccaggctgtagcgggagtctcccgcatagacgtcggtgaagtcgacgatcccggtgacctcggtcgcggccaggtccacgaagatgttggtcccgtgcaggtcgccgtggacgaaccggggttcgcggccggccagcagcgtgtccacgtccggcagccagtcctccaggcggtccagcagccggggcgagaggtagccccacccgcggtggtcctcgacggtcgccgcgcggcgttcccgcagcagttccgggaagacctcggaatggggggtgagcacggtgttcccggtcagcggcaccctgtgcagccggccgagcacccggccgagttcgcgggccagggcgagcagcgcgttccggtcggtcgtgccgtccatcgcggaccgccaggtggtgccggtcatccggctcatcaccaggtagggccacggccaggctccggtgccgggccgcagctcgccgcggccgaggaggcggggcaccggcaccggggcgtccgccaggaccgcgtacgcctccgactccgacgcgaggctctccggaccgcaccagtgctcgccgaacagcttgatcaccgggtcgggctcgccgaccagtacggggttggtgctctcgccgggcacccgcagcaccggcggcaccggcagcccgagctcctccagggctcggcgggccagcggctcccagaattcctggtcgttccgcaggctcgcgtaggaatcatccgaatcaatacggtcgagaagtaacagggattcttgtgtcacagcggacctctattcacagggtacgggccggcttaattccgcacggccggtcgcgacacggcctgtccgcaccgcggatcaggcgttgacgatgacgggctggtcggccacgtcggggacgacggggagtcaggcaactatggatgaacgaaatagacagatcgctgagataggtgcctcactgattaagcattggtaactgtcagaccaagtttactcatatatactttagattgatttaaaacttcatttttaatttaaaaggatctaggtgaagatcctttttgataatctcatgaccaaaatcccttaacgtgagttttcgttccactgagcgtcagaccccgtagaaaagatcaaaggatcttcttgagatcctttttttctgcgcgtaatctgctgcttgcaaacaaaaaaaccaccgctaccagcggtggtttgtttgccggatcaagagctaccaactctttttccgaaggtaactggcttcagcagagcgcagataccaaatactgtccttctagtgtagccgtagttaggccaccacttcaagaactctgtagcaccgcctacatacctcgctctgctaatcctgttaccagtggctgctgccagtggcgataagtcgtgtcttaccgggttggactcaagacgatagttaccggataaggcgcagcggtcgggctgaacggggggttcgtgcacacagcccagcttggagcgaacgacctacaccgaactgagatacctacagcgtgagctatgagaaagcgccacgcttcccgaagggagaaaggcggacaggtatccggtaagcggcagggtcggaacaggagagcgcacgagggagcttccagggggaaacgcctggtatctttatagtcctgtcgggtttcgccacctctgacttgagcgtcgatttttgtgatgctcgtcaggggggcggagcctatggaaaaacgccagcaacgcggcctttttacggttcctggccttttgctggccttttgctcacatgttctttcctgcgttatcccctgattctgtggataaccgtattaccgcctttgagtgagctgataccgctcgccgcagccgaacgaccgagcgcagcgagtcagtgagcgaggaagcggaagagcgcccaatacgcaaaccgcctctccccgcgcgttggccgattcattaatgcagctggcacgacaggtttcccgactggaaagcgggcagtgagcgcaacgcaattaatgtgagttagctcactcattaggcaccccaggctttacactttatgcttccggctcgtatgttgtgtggaattgtgagcggataacaatttcacacaggaaacagctatgaccatgattaccagatctggctcgcaccgcggtggcggccgc
